# Supplementary material for: Heterogeneous levels of delta-like 4 within a multinucleated niche cell maintains muscle stem cell diversity
Source: eLife. 2022 Dec 30;11:e68180. doi: 10.7554/eLife.68180 (PMC9803355; doi:10.7554/eLife.68180)
Supplement: Supplementary file 1. — This table includes the expression (as log2 values) of Notch ligands in postnatal day 3, postnatal day 7, and adult. Related to Figure 2—figure supplement 1. [file elife-68180-supp1.docx]

**Supplemental Table S1. Microarray expression of Notch ligands in p3, p7 and adult muscle fibers** **(log2 values).**

|  | **p3** | **p7** | **Adult** |
| --- | --- | --- | --- |
| **Dll1** | 7.179268 | 7.864147 | 7.530619 |
| **Dll3** | 5.568594 | 5.568594 | 5.568594 |
| **Dll4** | 7.124728 | 7.676524 | 9.269889 |
| **Jag1** | 8.456506 | 8.075812 | 8.670788 |
| **Jag2** | 9.312074 | 8.530521 | 9.012903 |

**Supplemental Table S1.** This table includes the expression (as log2 values) of Notch ligands in postnatal day 3, postnatal day 7 and adult.
