# Supplementary material for: Heterogeneous levels of delta-like 4 within a multinucleated niche cell maintains muscle stem cell diversity
Source: eLife. 2022 Dec 30;11:e68180. doi: 10.7554/eLife.68180 (PMC9803355; doi:10.7554/eLife.68180)
Supplement: Supplementary file 2. — This table includes the forward and reverse primer sequences of genes amplified by qRT-PCR. Related to Figure 2—figure supplement 1, Figures 5 and 7. [file elife-68180-supp2.docx]

**Supplemental Table S2. Primers used for Quantitative RT-PCR.**

| **Gene Name** | **Forward Sequence 5’ to 3’** | **Reverse Sequence 5’ to 3’** |
| --- | --- | --- |
| GAPDH | GGCAAAGTGGAGATTGTTGC | AATTTGCCGTGAGTGGAGTC |
| Pax7 | GTGGAATCAGAACCCGACCTC | GTAGTGGGTCCTCTCAAAGGC |
| MyoD | CCACTCCGGGACATAGACTTG | AAAAGCGCAGGTCTGGTGAG |
| Myogenin | GAGACATCCCCCTATTTCTACCA | GCTCAGTCCGCTCATAGCC |
| Dll1 | TTGGGCTTCTCTGGCTTCAAC | TCCACACACTTGGCACCGTTAG |
| Dll3 | TCGTACGTGTGCCCTTCC | TGCTCTCTCCAGGTTTCAATG |
| Dll4 | GGAACCTTCTCACTCAACATCC | CTCGTCTGTTCGCCAAATCT |
| JAG1 | TCTCTGACCCCTGCCATAAC | TTGAATCCATTCACCAGATCC |
| JAG2 | TCCTCCTGCTGCTTTGTGAT | TGTCAGGCAGGTCCCTTG |

**Supplemental Table S2.** This table includes the forward and reverse primer sequences of genes amplified by qRT-PCR.
